# Bile Cast Nephropathy Secondary to Hemophagocytic Lymphohistiocytosis With Liver Failure

**DOI:** 10.7759/cureus.10226

**Published:** 2020-09-03

**Authors:** Juan Jose Chango Azanza, Nerea Lopetegui Lia, Paola Michelle Calle Sarmiento

**Affiliations:** 1 Internal Medicine, University of Connecticut School of Medicine, Farmington, USA; 2 Internal Medicine, Catholic University of Cuenca, Cuenca, ECU

**Keywords:** bile salt nephropathy, cholemic nephropathy, hemophagocytic lymphohistiocytosis (hlh)

## Abstract

Acute kidney injury (AKI) is a common complication seen in patients with hemophagocytic lymphohistiocytosis (HLH). More than half of patients with HLH require renal replacement therapy (RRT). There are four main causes of kidney dysfunction in HLH, which include acute tubular necrosis (ATN), hypoperfusion, tumor lysis syndrome (TLS), and HLH-related glomerulopathies. Bile cast nephropathy (BCN) is a known cause of kidney injury in patients with liver failure and hyperbilirubinemia. We present the case of a 58-year-old man who presented to the hospital with painless jaundice, choluria, acholia, and generalized malaise and was found to have hyperbilirubinemia and kidney injury in the setting of HLH, who underwent a renal biopsy showing bile salt casts with degenerating tubular lining cells consistent with BCN. This case highlights the importance of considering BCN as a cause of kidney injury when a patient with HLH presents with liver failure and elevated bilirubin levels.

## Introduction

Hemophagocytic lymphohistiocytosis (HLH) is a rare and devastating disorder in which an uncontrolled activation of the immune system, in particular the hematophagocytic histiocytes, provokes a cytokine storm and multiorgan failure [1]. It has been recognized that acute kidney injury (AKI) can occur in HLH due to acute tubular necrosis (ATN) (49%), hypoperfusion (46%), tumor lysis syndrome (TLS) (29%), and HLH-related glomerulopathies (17%) [2]. However, other important cause of kidney injury in HLH could be bile cast nephropathy (BCN) or cholemic nephropathy. BCN has been described as a cause of kidney failure especially when there is liver injury accompanied by high levels of bilirubins. However, to our knowledge, BCN in the setting of liver injury secondary to HLH has not been reported. BCN has been described as a consequence of liver dysfunction with or without the presence of cirrhosis and hepatorenal syndrome (HRS) [3]. We present a case describing clinical and pathologic evidence of BCN in a patient with liver failure in the setting of HLH and the relevance of this underrecognized cause of AKI.

## Case presentation

**Figure 1:**
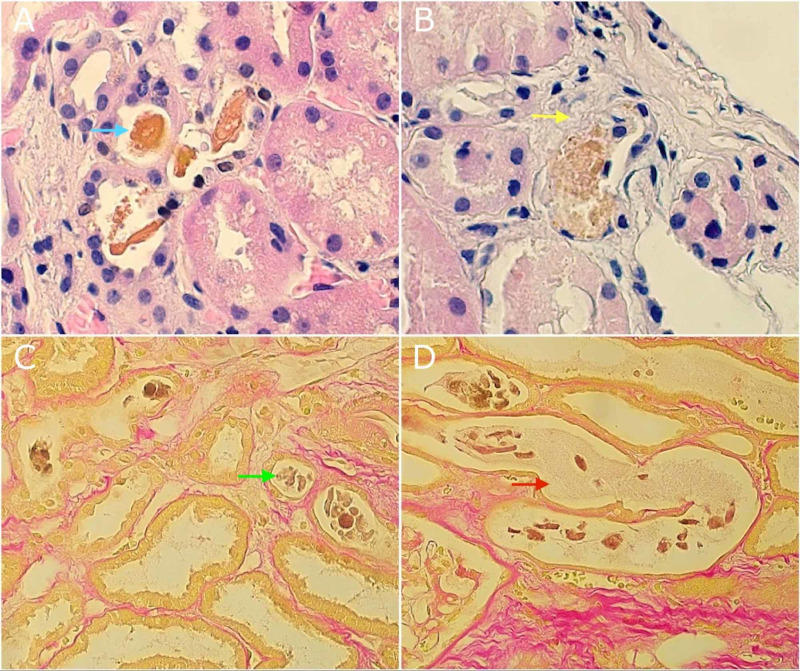
Kidney Biopsy Light Microscopy Haemotoxylin and eosin (H&E) stain showing bile stained casts (blue arrow) (A) and a bile stained cast with surrounding degenerating tubular lining cells (yellow arrow) (B). Hall's stain showing cast material positive for bile (green arrow) (C) and cast fragments positive for bile (red arrow) (D).

A 58-year-old man with an unremarkable past medical history presented to the hospital due to a five-day history of painless jaundice accompanied by fatigue, generalized weakness, choluria, and acholia. His vital signs were within normal limits. On physical examination, he exhibited generalized jaundice. His laboratory workup showed leukocytosis of 23,000 × 10^3^/µL (normal value 3.8-10.6 × 10^3^/µL), hemoglobin 10.5 g/dL (normal value 13-18 g/dL), creatinine 2.5 mg/dL with unknown baseline (normal value 0.6-1.2 mg/dL), aspartate aminotransferase (AST) 1,167 U/L (normal value 17-35 U/L), alanine aminotransferase (ALT) 1,447 U/L (normal value 8-39 U/L), alkaline phosphatase 215 U/L (normal value 39-113 U/L), total bilirubin 52.7 mg/dL (normal value 0.1-1.2 mg/dL) with direct bilirubin >30 mg/dL (normal value 0.0-0.5 mg/dL), international normalized ratio (INR) 1.2 (normal ratio 0.8-1.1), albumin 3.8 g/dL (normal value 3.8-5.4 g/dL), ferritin 11,559 ng/mL (normal value 16-336 ng/mL), and triglycerides 380 mg/dL (normal value <150 mg/dL). A urinalysis showed granular casts with no signs of infection. Imaging studies including an MRI of the abdomen, magnetic resonance cholangiopancreatography (MCRP), and endoscopic ultrasonography of the liver (EUS) showed diffuse liver inflammatory changes, periportal and peripancreatic lymphadenopathies, and no obstructions or dilatations of the biliary tract. A liver biopsy showed severe cholestatic hepatitis, periportal hemosiderin deposition, and a large number of plasma cells in the periportal areas suspicious for autoimmune hepatitis and cholangitis. Due to kidney failure, he underwent a renal biopsy revealing bile salt casts with a positive Hall's stain and degenerating tubular lining cells consistent with BCN (Figure 1).

Extensive workup for infection, autoimmunity, metabolic disorders, and hemochromatosis (including negative genetic mutations) was unrevealing. A bone marrow biopsy showed rare hemophagocytosis and hemolysis but was non-diagnostic. He required hemodialysis for renal failure. He was treated with systemic corticosteroids with significant improvement and subsequently discharged from the hospital after multiple days of treatment. The patient was readmitted months later and found to have encephalopathy, fevers, pancytopenia, elevated ferritin (above 40,000 ng/mL), hypertriglyceridemia, and splenomegaly (14.7 cm). His soluble interleukin-2 receptor level was increased significantly to 44,176 pg/mL (normal value 175-858 pg/mL). A second bone marrow biopsy was done showing hypocellularity, plasmacytosis, and hemophagocytosis consistent with HLH. Therefore, he met clinical and laboratory criteria for HLH. The patient was started on treatment with etoposide. Unfortunately, he decompensated and developed worsening liver failure. His family decided to transition to comfort care, and the patient expired several days later.

## Discussion

HLH is a rare and devastating disorder in which an uncontrolled immune activation causes a severe inflammatory syndrome [1]. A defect in the natural killer (NK) cells and cytotoxic T lymphocytes (CTLs) results in the proliferation of the hemophagocytic histiocytes (macrophages) [4]. The inability of NK cells and CTLs to destroy target cells can be inherited (primary) or acquired (secondary) due to an antigenic challenge from infection, malignancy, autoimmunity, and other causes [5]. Genetic forms of HLH exhibit defects in the ability of NK cells and CTLs to process and transport cytotoxic granules. The activation of NK cells and CTLs provokes a cascade of inflammatory effects. The increased production of interferon-gamma plays a significant role in the activation of benign macrophages in multiple organs, such as the spleen, liver, lymph nodes, and others. Other important cytokines are the tumor necrosis factor-alpha (TNF-α) and interleukins 1, 6, and 8 [6]. The proliferating macrophages (histiocytes) start engulfing the hematopoietic cells, such as red blood cells, white blood cells, and platelets in a process termed hemophagocytosis [4]. The consequence of HLH is a cytokine storm that causes a severe systemic inflammatory response syndrome that leads to multiorgan failure with high mortality (ranging from 22% to 88%) [7]. 

HLH can manifest in single or multiple episodes (especially primary HLH) usually triggered by a defined insult. The most common infectious trigger is a viral infection, especially due to the Epstein-Barr virus [8]. Multiple autoimmune conditions have been associated with HLH including systemic lupus erythematosus, juvenile idiopathic arthritis, adult-onset Still’s disease, among others [9]. Malignancy (especially lymphoma), immunodeficiency, and metabolic conditions are also known triggers of HLH. HLH mostly affects children but adult cases are increasing in the last 10 years [1]. The incidence of HLH in adults is not known due to its rarity. The clinical manifestations of HLH are diverse and non-specific. There is an overlap of clinical features with multiple other syndromes. Therefore, a high index of suspicion is required for its diagnosis.

The Histiocyte Society (HLH-2004 trial) proposed diagnostic criteria for the identification of HLH [1]. The criteria include molecular testing consistent with HLH or five out of eight of the following criteria: fever (frequently found in HLH), cytopenias (affecting at least two lineages), splenomegaly, hyperferritinemia, hypertriglyceridemia and/or hypofibrinogenemia, impaired NK cell function, elevated soluble cluster of differentiation 25 (sCD25) also known as soluble interleukin 2 receptor (sIL2R), and evidence of hemophagocytosis (in the spleen, lymph nodes or bone marrow). It is important to recognize that these diagnostic criteria were developed for children and with a focus on research. However, there are no universally accepted criteria for the diagnosis of HLH in adults, and the diagnostic criteria from the Histiocyte Society are now widely used [1,10]. Other findings in HLH include transaminitis, hyperbilirubinemia, coagulopathy, hyponatremia, edema, rash, hypoalbuminemia, elevated lactate dehydrogenase, neurologic (altered, mental status, seizures, focal deficits, increased cell count and protein content in the cerebrospinal fluid), and others [1]. 

AKI is common in HLH with an incidence of 62% and a renal replacement therapy (RRT) requirement of 59% [2]. The causes of AKI in HLH include ATN (49%), hypoperfusion (46%), tumor lysis syndrome (29%), or HLH-associated glomerulopathies (17%). Among the causes of ATN are ischemic lesions, nephrotoxic drugs, and/or high levels of TNF-α [11]. Nephrotic syndrome has been reported and is associated with collapsing glomerulopathy, minimal change glomerulopathy, and thrombotic microangiopathy. HLH could cause direct interstitial and tubular infiltration of activated macrophages and T lymphocytes. However, finding infiltration of activated macrophages and T lymphocytes is uncommon and may have less strength in the causation of kidney injury [2].

Interestingly, AKI in HLH due to BCN has not been reported. BCN is a known cause of AKI and occurs mostly in patients with liver disease. HRS is a common cause of AKI in cirrhotic patients [3]. Furthermore, non-HRS AKI can be seen in patients with inflammation, bacterial translocation, cardiac dysfunction, and BCN [12]. BCN or cholemic nephropathy occurs in patients with a liver injury with hyperbilirubinemia [3]. Histologic findings in BCN include the observation of bilirubin casts identified by Hall’s stain within the tubular lumen or massive pigment inclusions in the tubular epithelial cells, and tubular epithelial damage. These findings were present in the kidney biopsy of our patients and correlated with the liver injury from HLH with profound hyperbilirubinemia. Therefore, considering the liver involvement of HLH as a plausible cause of AKI seems appropriate and it should be suspected especially if the patient also has hyperbilirubinemia.

Treatment for HLH focuses on halting the underlying trigger and hyperactive immune system. Treatment of a suspected malignancy or infection should be started without delays [1]. Treatment options are chemoimmunotherapy (etoposide, corticosteroids), treatment of underlying conditions, and bone marrow transplantation in selected cases [4]. Treatment of the renal dysfunction in HLH in addition to these measures is supportive. RRT is frequently needed, and it might play a role in the clearance of hyperbilirubinemia in the liver failure caused by HLH. 

## Conclusions

BCN occurs in patients with liver failure and hyperbilirubinemia, which is commonly seen in patients with HLH. It may occur mostly when patients have an elevated AST level and profound hyperbilirubinemia. Diagnosis is usually made by renal biopsy, making it challenging to recognize without a high clinical suspicion. Therefore, we hypothesize that BCN is an important contributor to the kidney injury seen in HLL. RRT is usually required and may play an important role in the removal of bilirubin.
